# Clinical Presentation of a COVID-19 Delta Variant Patient: Case Report and Literature Review

**DOI:** 10.7759/cureus.18603

**Published:** 2021-10-08

**Authors:** Farah Chohan, Angela Ishak, Tyler Alderette, Pedram Rad, George Michel

**Affiliations:** 1 Internal Medicine, Larkin Community Hospital, South Miami, USA; 2 Research and Academic Affairs, Larkin Community Hospital, South Miami, USA; 3 Psychiatry, Larkin Community Hospital, South Miami, USA

**Keywords:** spike glycoprotein, mutation, immune escape, genetic variation, sars-cov-2, tocilizumab, pandemic, delta variant, coronavirus, covid-19

## Abstract

The severe acute respiratory syndrome coronavirus 2 (SARS-CoV-2) causes severe bilateral pneumonia and acute respiratory distress syndrome (ARDS) which can lead to difficulty breathing. Many cases require mechanical ventilation and intensive care unit management. The need for mechanical ventilation and ICU admission seems to be more evident in patients that were unvaccinated for COVID-19 at the time of admission. We discuss a case of a 63-year-old African-American woman who presented as a transfer to our hospital facility with acute hypoxic respiratory failure. She was already intubated and mechanically ventilated prior to her transfer. She had a one-week history of shortness of breath and cough productive of white, blood-tinged sputum. A two-day history of diarrhea was also reported before admission to the other hospital where she stayed for a week before transfer to our intensive care unit. She had no significant past medical history and was unvaccinated for COVID-19, and was suspected to be infected with the Delta strain of COVID-19. Her primary diagnosis at admission was COVID pneumonia and acute hypoxic respiratory failure. Her condition worsened over a period of one week. Chest X-Ray, at the time of arrival, showed bilateral patchy opacities consistent with COVID-19 pneumonia. After an extensive review of her labs and reports, the patient was attributed to be at a high risk for acute decompensation (or catastrophically ill), thus requiring critical care management. Over a course of 12 days, she was aggressively treated with antibiotics, steroids, remdesivir and tocilizumab. Her condition gradually deteriorated and she eventually passed away. It can be noted that most of the severe cases, especially ICU admissions, comprise people who are unvaccinated. We can safely conclude that although vaccination may not prevent re-infection, it does result in better clinical outcomes.

## Introduction

The emergence of severe acute respiratory syndrome coronavirus 2 (SARS-CoV-2) in late 2019 led to a global pandemic healthcare crisis across the world resulting in more than 210 million cases worldwide and 4.41 million deaths to date. Following a period of 11-month evolutionary stagnation, there has been an emergence of SARS-CoV-2 sets of mutations, termed as "variants of concern" by the US Department of Health and Human Services. The B.1.1.7 (Alpha), B.1.351 (Beta), P.1 (Gamma), and B.1.617.2 (Delta) variants are all considered the “variants of concern,” which means a variant with one or more mutations affecting virus characteristics which include transmissibility and antigenicity [1].

The Delta variant is characterized by mutations in the spike proteins, particularly in the lineage of B.1.617, and this mutation has led the virus to become more transmissible than proviso variants (40-60% more than the alpha variant) [2]. In the US, the Delta variant was first identified in March 2021. Since then, it has seemingly become the predominant variant in the US and has caused a massive wave of new infections, especially in the Southeastern states of the US, which may partly be due to the low vaccination rates. Reports have shown substantially higher numbers of cases, hospitalizations, and deaths among areas with low vaccination rates and health mitigation efforts [3].

The Delta variant also causes more severe disease and is less susceptible to vaccines than previous lineages [4]. The symptoms resemble the common cold such as rhinorrhea, fever, and pharyngitis, and it is less likely to cause loss of smell when compared with other variants such as the alpha variant [5]. 

Several countries have reported that the Delta variant has a significantly higher risk of hospitalization, ICU admission, developing pneumonia, and about 137% greater risk of death compared to non-variant SARS-CoV-2 [6]. Public Health England performed a study to compare vaccinated (with Pfizer-BioNTech and AstraZeneca-Oxford) and unvaccinated people against the Delta variant. Their study showed that those fully vaccinated had more than 30% fewer cases of symptomatic disease compared to those who are unvaccinated [7]. Moreover, a study conducted by researchers at the Imperial College, London, UK reported that the prevalence among unvaccinated people was three-fold higher and had higher rates of infection [8].

We present a case on a COVID-19 Delta variant patient. We discuss the progression of the disease in a previously healthy and unvaccinated patient and the effectiveness of the currently approved treatments.

## Case presentation

Clinical course

A 63-year-old African-American woman presented to the hospital from the Doctor’s Hospital in The Bahamas with a one-week history of shortness of breath and productive cough of white, blood-tinged sputum, and a two-day history of diarrhea before admission. She had no significant past medical history.

The patient tested positive for COVID-19 via RT-PCR (reverse transcription-polymerase chain reaction) on July 17th. In the previous facility, the patient was initially placed on the face mask and was upgraded to a high-flow nasal cannula. However, the patient soon desaturated and experienced respiratory arrest. Therefore, the patient was intubated for a couple of days and then transferred to our facility. Prior to admission, she was initially administered ceftriaxone 1 g and azithromycin 500 mg, IV daily. After intubation, she was started on dexamethasone, remdesivir, piperacillin/tazobactam, levofloxacin, and mechanical ventilation were to be continued in the following days. She was therefore transported to our facility via an air ambulance and admitted to the ICU. Her qSOFA (quick sequential organ failure assessment) score was 2 (considered high-risk), SOFA was 8 points, mortality thought to be <33%, APACHE II (acute physiology and chronic health evaluation) suggested 30% estimated postoperative mortality. After an extensive review of all her labs and reports, the patient was attributed to be at a high risk for acute decompensation (or catastrophically ill) thus requiring critical care management. 

On July 26th, she developed deep vein thrombosis (DVT). On July 27th, the patient started developing signs of kidney failure with a drop in glomerular filtration rate (GFR) to 19. She also desaturated several times that night but her saturation returned to 99% the following morning. On July 31st, the patient developed disseminated intravascular coagulation (DIC) and her labs showed a drop in hemoglobin levels from 9.2 to 7.2. The patient was able to recover with appropriate treatment, however, she subsequently developed respiratory failure the following day with an oxygen saturation of 51% while remaining anuric. Her SOFA score at this time correlated to more than 90% mortality. Appropriate measures were taken and her saturation improved to 90%. Her clinical course kept deteriorating with further episodes of respiratory failure and two episodes of cardiac arrest on August 3rd to 6th. Finally, on August 8th, the patient suffered another cardiac arrest and developed pulseless electrical activity; however, attempts to resuscitate her were unsuccessful and therefore the patient died. 

Imaging and laboratory tests

**Figure 1:**
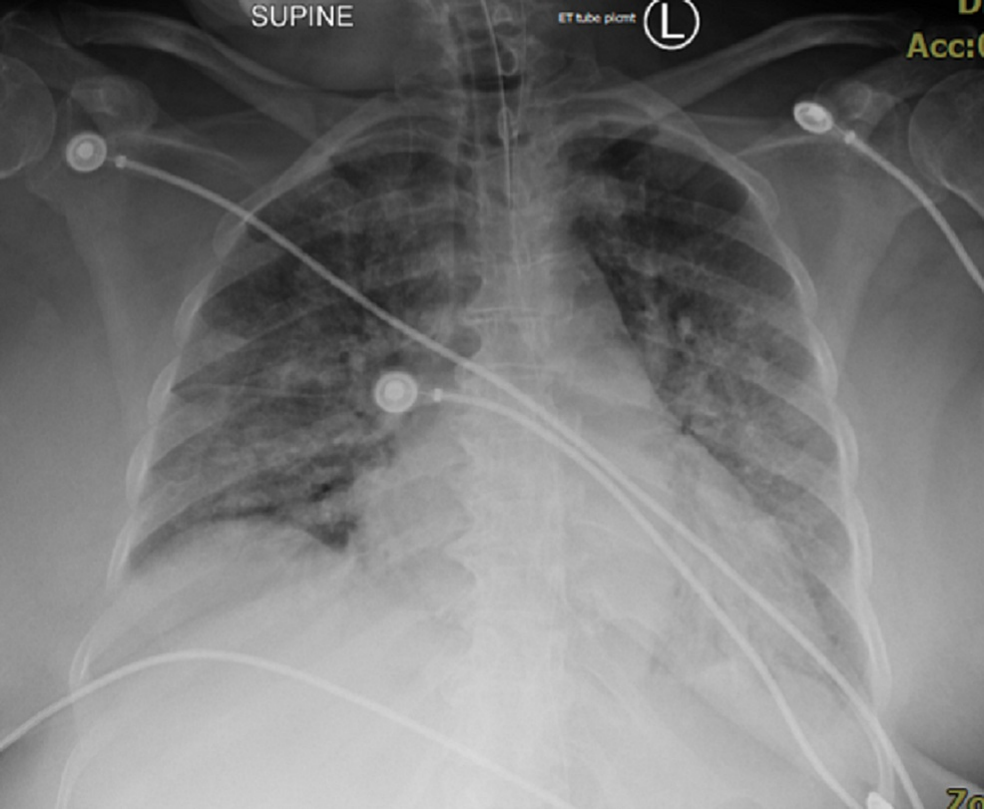
Bilateral patchy opacities consistent with COVID-19 pneumonia

**Figure 2:**
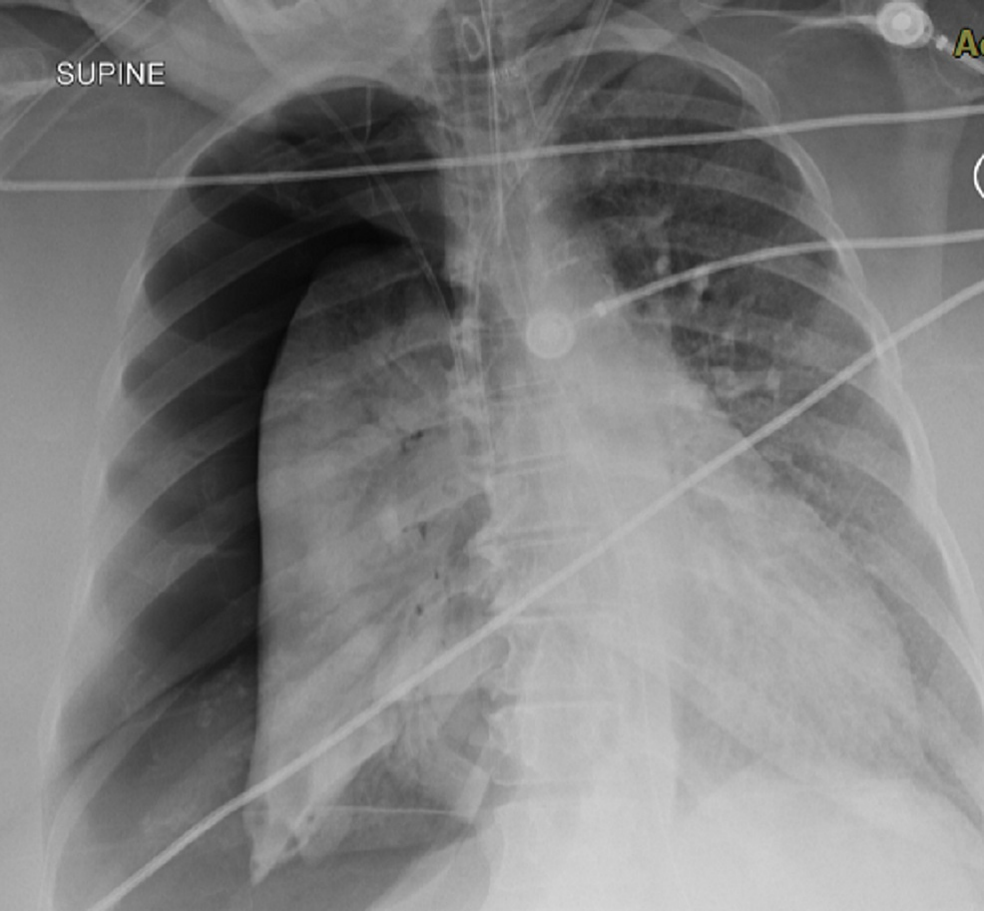
Pneumothorax seen on the right side

On July 25th, the patient underwent a chest X-ray that showed bilateral patchy opacities consistent with COVID-19 pneumonia (Figure 1). Her labs were significant for neutrophilic leukocytosis, hypernatremia, hyperglycemia, thrombocytosis, normocytic anemia, and elevated inflammatory markers (D-Dimer 768, above 630 (age-adjusted D-Dimer), Well's score 2.5 points). On July 26th, an ultrasound revealed a non-obstructive DVT on the right common femoral vein and a CT scan showed diffuse bilateral mixed ground-glass and consolidative opacities, compatible with atypical pneumonia, with superimposed pulmonary edema. Some underlying nodular opacities were also seen. There was an enlarged right upper paratracheal lymph node which was likely reactive. July 27th, her creatine increased from 0.69 to 2.46, BUN (blood urea nitrogen) from 21 to 49, and GFR dropped to 19. August 3rd, DIC workup was positive (DIC workup positive: INR 3.8, PT 44.2, D-Dimer 3683, fibrinogen 180) and heparin-induced thrombocytopenia (HIT) antibodies were negative. On August 6th, the chest X-ray showed pneumothorax (Figure 2). 

Interventions

Mechanical Ventilation and Oxygen Therapy

Since arrival, the patient has been treated with mechanical ventilation. The initial settings were AC/PC, RR 30, Pi 25, Ti 1.0, PEEP 12, FiO2 67%. The FiO2 was increased to 80% on day 3 as the patient kept desaturating. This initially helped maintain an O2 saturation of 99%. However, she still developed respiratory failure and required removal from the ventilator and Bag-valve mask for further ventilation on day 8. Once saturation reached 80%, the patient was placed on mechanical ventilation again. Settings were adjusted to pressure control and inverse ratio ventilation was employed (I:E 2.5:1) to improve oxygenation. PEEP was also increased for further recruitment. Saturation subsequently improved to 90%. On day 10, ventilator settings were adjusted to AC/PC 30, Pi 33, Ti 1.60, PEEP 20, FiO2 100% as her pO2 decreased to 60% overnight. The patient remained on mechanical ventilation until she died. 

Plasma Exchange

On the third day of hospitalization, the patient was started on convalescent plasma. 

Antiviral Therapy

The patient was started on remdesivir on day 1 where she received three doses. She received another four doses, two-by-two doses on days 2 and 3 respectively. 

Antibiotic Therapy

On admission, the initial antibiotic therapy given by the other facility was changed to cefepime, doxycycline, and vancomycin for 5 days. Due to the worsening kidney function vancomycin was discontinued and replaced with linezolid 600 mg every 12 hours on day 3. On day 4, meropenem IV was added to her antibiotic regimen. Cefepime and doxycycline courses were completed on day 5, linezolid course was completed on day 8, and meropenem course was completed on day 12.

Glucocorticoid Therapy

On admission, the patient was started on dexamethasone 10 mg BID IV. On day 2, dexamethasone was increased from twice daily to every 8 hours. On day 7, the dose was reduced to 6 mg and was now given every 12 hours daily until the patient was deceased. 

Anticoagulation Therapy

The patient’s D-dimer was elevated throughout hospitalization and she was diagnosed with DVT on day 2. Heparin 7 unit/Kg/h IV was therefore started, however, this was stopped on day 7 as the patient started showing clinical signs of DIC. An inferior vena cava (IVC) filter was placed on day 9 to prevent further blood clotting and the patient was started on argatroban 50 mg. 

Renal Function Management

The patient’s kidney function began deteriorating on day 3 of hospitalization and she developed anuria on day 5. Therefore, on day 5, the patient was administered 80 mg of furosemide and a trialysis catheter was placed for dialysis. On day 6, dialysis was started for an hour and removed 1 liter of fluids, however, the patient turned hypotensive and dialysis was stopped. On day 7, bumetanide 1 mg twice daily as the patient in addition to another round of dialysis. The patient kept deteriorating and received dialysis for 1 hour daily from day 8 and onwards until she died. 

Other Therapies

Sedation with propofol 30ml/h IV and analgesia with 50 mcg/h IV were administered daily. The patient was also administered a single dose of tocilizumab, an Il-6 antagonist monoclonal antibody, on day 2. Additionally, the patient was administered a single dose of sevelamer 1600 mg due to hyperphosphatemia of 12.1 on day 9. 

## Discussion

In this case report, we discuss the clinical course of an unvaccinated patient who is thought to have been infected with the Delta variant of COVID-19. The Delta variant has turned parts of Florida into coronavirus hot spots, and the curve looks nowhere close to reaching a plateau or showing a downward trend. This highly contagious strain now accounts for one in five COVID-19 patients hospitalized nationwide. The Florida Department of Health released new data showing 150,118 new cases of COVID-19 within the state over the past week. In all, Florida has recorded at least 2,994,019 confirmed COVID cases statewide and 41,937 deaths. Keeping in mind the aforementioned statistics, the patient’s travel history, clinical presentation, and rapid deterioration of her condition, we assume that she was infected with the Delta variant. At the time of arrival, she was already being intubated and mechanically ventilated. The management of this patient was guided using several prognostic scores (qSOFA, SOFA, and APACHE II) which all indicated that the patient was severely ill. Therefore, the treatment options that we used for this patient were the ones that have shown improved clinical outcomes in other critically ill patients. The main interventions employed for this patient included mechanical ventilation and oxygen therapy, plasma exchange therapy, antimicrobial therapy, glucocorticoid therapy, anticoagulation therapy, immunomodulating therapy, and management of the deteriorating kidney function.

There is debate in the literature in regards to the correct timing to start mechanical ventilation and which settings are the most optimal in severe COVID-19 [9,10]. The patient arrived at our faculty already intubated and mechanically ventilated due to several episodes of respiratory arrest. Due to the lack of clear evidence on which settings should be followed for COVID-19 pneumonia, the American Thoracic Society guidelines were used as a reference [11]. The ventilation settings were adjusted appropriately as the patient’s clinical course deteriorated. 

Acute kidney injury has been associated heavily with COVID-19 infection [12]. However, there is no effective treatment and therefore all that could be offered for critically ill patients such as ours is supportive treatment with dialysis and diuretics [13]. Additionally, DIC and blood clots are also common complications in COVID-19 patients [14]. Heparin is thought to be effective in treating and preventing these complications [15], however, due to the patient’s worsening kidney function, she had to be switched to argobratan and an IVC filter needed to be placed.

There are various monoclonal antibodies being investigated for their efficacy in COVID-19 management. Another case report which reported a Delta variant breakthrough infection in a vaccinated patient showed full recovery after treatment with bamlanivimab/etesevimab, however, the patient was not critically ill [16]. There is also evidence that tocilizumab could be efficacious in reducing mortality and the need for mechanical ventilation in COVID-19 patients [17]. Therefore a trial of single-dose tocilizumab was given to our patient.

## Conclusions

This case highlights that the currently available treatments are still not effective in severe COVID-19 infection. It has been becoming increasingly evident that although breakthrough infections are occurring with the Delta variant, the currently available vaccines remain the most effective protection against severe disease, hospitalization, and death. The overwhelming majority of severe cases occurring in the US are among unvaccinated individuals. Deepening the study of spike protein mutations will help to better understand how to combat COVID-19 and its variants.
